# Confronting urethrorrhagia after Otis urethrotomy: a case report

**DOI:** 10.1186/s13256-023-04261-w

**Published:** 2023-12-07

**Authors:** Uttam Kumar Mete, Rohit Sanjay Deshpande

**Affiliations:** Department of Urology, PGIMER, Chandigarh, 12 India

**Keywords:** Otis, Urethrotomy, Angioembolization, Urethrorrhagia

## Abstract

**Background:**

Otis urethrotomy can sometimes lead to troublesome bleeding after seemingly uneventful procedures. This case report highlights one such case which went unnoticed initially; the bleeding was erroneously ascribed to the prostate, thereby falsely indicting the “decoy” prostate.

**Case presentation:**

A 78-year-old Asian gentleman was referred to our hospital with complaint of intractable bleeding after undergoing laser enucleation of prostate at another institute, wherein he further underwent unsuccessful bilateral angioembolization of pudendal arteries. On endoscopy (for hemostasis), we found a spurting vessel in the navicular fossa, which was effectively controlled.

**Conclusions:**

This case report highlights the importance of performing prompt endoscopy in case of uncontrolled bleeding after prostate endoscopic surgery.

## Background

Otis urethrotomy occasionally results in uncontrollable bleeding after seemingly straightforward surgeries. This case report focuses on one such instance that initially went undiscovered; the bleeding was mistakenly attributed to the prostate, and was swiftly taken care of by a simple procedure.

## Case presentation

A 78-year-old Asian gentleman (who was on dual anti-platelet drugs, in addition to being diabetic and hypertensive) underwent laser anatomic enucleation of prostate for benign enlargement of the prostate, at another institute. The patient was off anti-platelet medications for 1 week prior to enucleation surgery. The patient had per-urethral bleeding (without the initiation of anti-platelet medications) starting from the 3rd post-operative day (as soon as the 22 French tri-way Foley’s catheter was removed), and was re-admitted at the same institute; penile jacketing was done, perineal compression was given and he received 4 blood transfusions. Despite such conservative measures, the bleeding continued intermittently, and the patient underwent bilateral angioembolization of the internal pudendal arteries along with unilateral angioembolization of a bulbar artery. However, the bleeding continued intermittently and the patient was then referred to our institute.

**Fig. 1:**
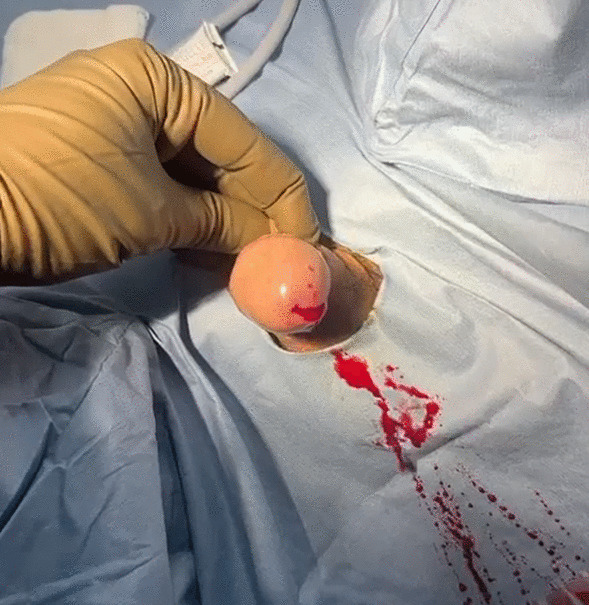
Fresh bleeding seen (prior to inserting the cystoscope) on the operating table, emanating from the urethra, with obvious spurting of blood

**Fig. 2:**
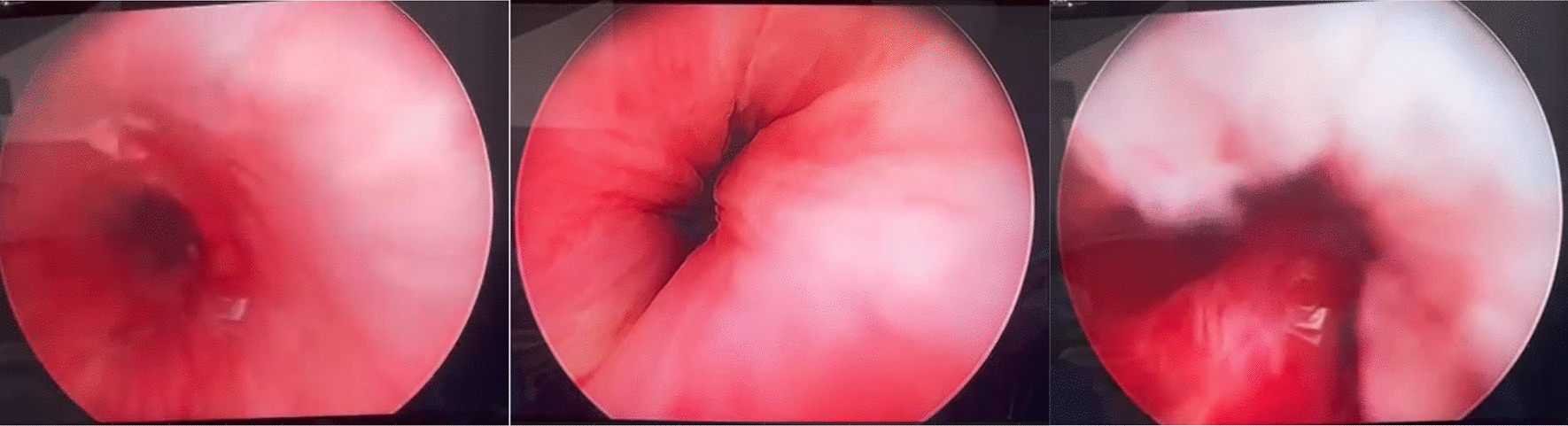
Images showing normal bulbar and penile urethra, and normal prostatic fossa. No evidence of bleeding was found from these sites

**Fig. 3:**
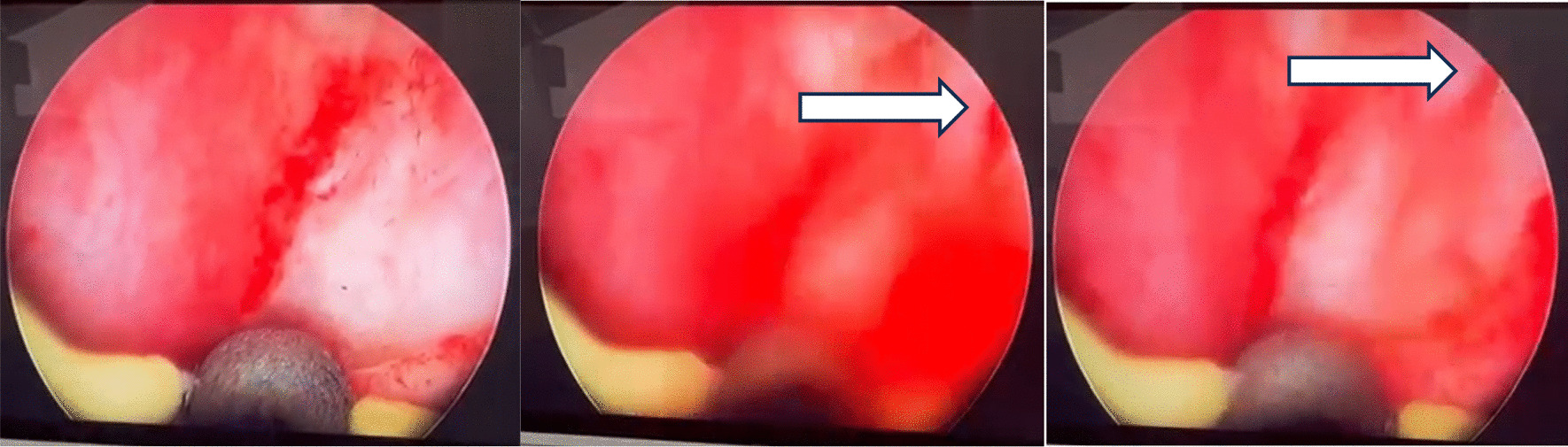
While withdrawing the scope, we found this actively spurting vessel (shown by the white arrow) in the fossa navicularis

**Fig. 4:**
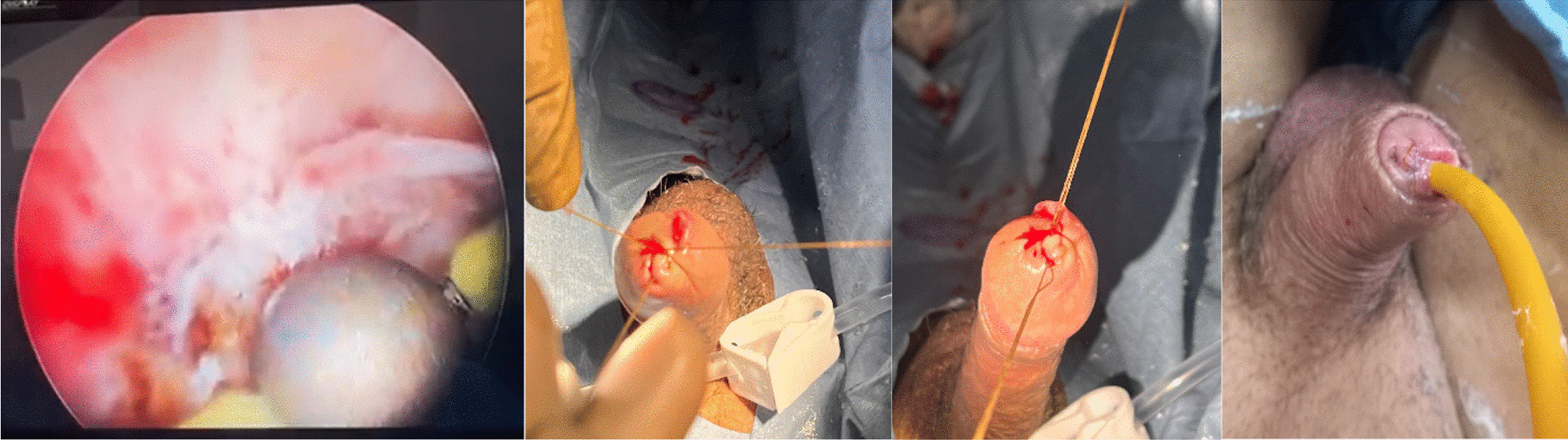
The vessel was fulgurated using a roller-ball attached to monopolar electrocautery, with a 30-degree telescope and 26 French sheath. Glycine was used as the irrigating fluid. Also, a suture was taken over the glans (with the suture knot outside), incorporating the site of the bleeding vessel, taking care that the meatus was not stenosed (allowing enough space for insertion of an 18 French Foley’s catheter)

After optimizing the patient with resuscitative intravenous fluids, we took the patient for cystoscopy (anti-platelet drugs were still kept on hold), under spinal anesthesia (see Figs. 1, 2, 3, 4).

After controlling the bleeding, the patient was kept off antiplatelet drugs for 7 days, after which they were restarted. Foley’s catheter was removed on the 2nd post-operative day, and the patient had an uneventful recovery.

## Discussion

Hemorrhage in the resected area is a common complication both during and after TURP, and every effort should be made to achieve hemostasis during the operation, to prevent the need for a return to the operating room. The Veterans Affairs Cooperative Study of 3885 patients found a transfusion rate of 2.5% [1]. Other early data reported high transfusion rates with over 20% of patients receiving transfusion [2], and more recent data from an analysis of RCTs found that 4.4% of patients would require transfusion [3].

Urethrorrhagia, a rare urological disorder, is described as continuous or intermittent active urethral bleeding irrespective of urination. Massive bleeds following internal urethrotomy have been described in literature, sometimes resulting in bulbourethral artery pseudoaneurysm [4]. Severe urethrorrhagia following traumatic catheterization (resulting in pseudoaneurysm of distal branches of the internal pudendal artery, requiring internal pudendal artery angioembolization) has also been described [5]. Deep cuts into the corpus spongiosum and, occasionally, the corpora cavernosa through the scar tissue are the most common causes of bleeding after OIU. Most of the time, mild bleeding stops on its own or with external compression. Urethrorrhagia following OTIS urethrotomy is considered by some as evidence of deep urethrotomy, and has even been held responsible for ensuing fibrotic process leading to ventral penile curvature [6].

**Fig. 5:**
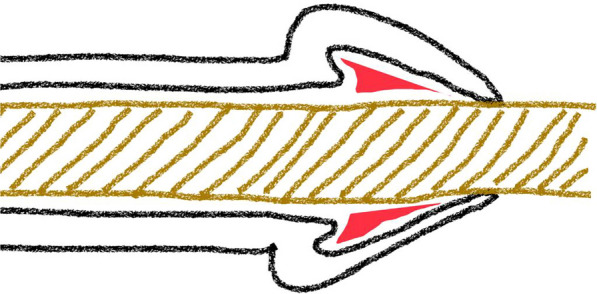
Red-shaded area represents the navicular fossa, in which smaller lumen catheters (depicted by the brown-colored tube traversing the urethral lumen) are less likely to exert the catheter-induced tamponade effect on urethral bleeding

Managing bleeding from the fossa navicularis can be challenging, since endoscopically, it is very difficult to gain access to an area which is situated near the tip of the penis (the irrigant also is unable to effectively distend this part of the urethra, due to continuously being drained through the external urethral meatus; hence vision is compromised). We suggest that the navicular fossa (the site of bleeding in the index case), being anatomically more capacious than the penile urethra, is ineffectively compressed especially by smaller-lumen Foley’s catheters (leading to loss of the tamponade effect of per-urethral catheters in this region) (as shown in Fig. 5).

## Conclusion

Urethrorrhagia is an uncommon and unexpected complication after TURP/prostate enucleation; in this case it was attributable to the ancillary procedure of an Otis urethrotomy. Endoscopy should be the initial step in the management approach of uncontrollable bleeding after prostate surgery. Angioembolization can prove futile in the management of bleeding due to Otis urethrotomy, and simpler alternatives must be attempted.

## Data Availability

Data sharing not applicable to this article as no datasets were generated or analysed during the current study.

## Abbreviations

RCT: Randomized controlled trial
TURP: Transurethral resection of prostate
OIU: Optical internal urethrotomy
